# Association of Electronic Cigarette Use With Smoking Habits, Demographic Factors, and Respiratory Symptoms

**DOI:** 10.1001/jamanetworkopen.2018.0789

**Published:** 2018-07-20

**Authors:** Linnea Hedman, Helena Backman, Caroline Stridsman, Jenny A. Bosson, Magnus Lundbäck, Anne Lindberg, Eva Rönmark, Linda Ekerljung

**Affiliations:** 1 The Obstructive Lung Disease in Northern Sweden Unit, Department of Public Health and Clinical Medicine, Occupational and Environmental Medicine, Umeå University, Umeå, Sweden; 2 Division of Nursing, Department of Health Sciences, Luleå University of Technology, Luleå, Sweden; 3 Division of Respiratory Medicine, Department of Public Health and Clinical Medicine, Umeå University, Umeå, Sweden; 4 Karolinska Institutet, Division of Cardiovascular Medicine, Department of Clinical Sciences, Danderyd University Hospital, Stockholm, Sweden; 5 The Obstructive Lung Disease in Northern Sweden Unit, Division of Medicine, Department of Public Health and Clinical Medicine, Umeå University, Umeå, Sweden; 6 Krefting Research Center, Sahlgrenska Academy, University of Gothenburg, Gothenburg, Sweden

## Abstract

**Importance:**

There is an ongoing debate about whether electronic cigarettes (e-cigarettes) are the solution to the tobacco epidemic or a new public health threat. Large representative studies are needed to study e-cigarette use in the general population, but hardly any have been published.

**Objectives:**

To estimate the prevalence of e-cigarette use and to investigate the association of e-cigarette use with smoking habits, demographic factors, and respiratory symptoms.

**Design, Setting, and Participants:**

Cross-sectional, population-based study of random samples of the population, performed within the Obstructive Lung Disease in Northern Sweden (OLIN) study and West Sweden Asthma Study (WSAS). The same validated questionnaire including identical questions was used in OLIN and WSAS. In 2016, OLIN and WSAS conducted postal questionnaire surveys in random samples of adults aged 20 to 75 years. In OLIN, 6519 participated (response rate, 56.4%); in WSAS, 23 753 participated (response rate, 50.1%).

**Main Outcomes and Measures:**

Electronic cigarette use, smoking habits, and respiratory symptoms.

**Results:**

Of 30 272 participants (16 325 women [53.9%]), 3897 (12.9%) were aged 20 to 29 years; 4242 (14.0%), 30 to 39 years; 5082 (16.8%), 40 to 49 years; 6052 (20.0%), 50 to 59 years; 6628 (21.9%), 60 to 69 years; and 4371 (14.4%), 70 to 75 years. The number of current smokers was 3694 (12.3%), and 7305 (24.4%) were former smokers. The number of e-cigarette users was 529 (2.0%), and e-cigarette use was more common among men (275 of 12 347 [2.2%; 95% CI, 2.0%-2.5%]) than women (254 of 14 022 [1.8%; 95% CI, 1.6%-2.0%]). Among current smokers, 350 of 3566 (9.8%; 95% CI, 8.8%-10.8%) used e-cigarettes compared with 79 of 6875 (1.1%; 95% CI, 0.9%-1.3%) in former smokers and 96 of 15 832 (0.6%; 95% CI, 0.5%-0.7%) in nonsmokers (*P* < .001). Among e-cigarette users who answered the survey question about cigarette-smoking habits (n = 525), 350 (66.7%; 95% CI, 62.7%-70.7%) were current smokers, 79 (15.0%; 95% CI, 11.9%-18.1%) were former smokers, and 96 (18.3%; 95% CI, 15.0%-21.6%) were nonsmokers (*P* < .001 for trend). In a regression analysis, e-cigarette use was associated with male sex (odds ratio [OR], 1.35; 95% CI, 1.12-1.62); age groups 20 to 29 years (OR, 2.77; 95% CI, 1.90-4.05), 30 to 39 years (OR, 2.27; 95% CI, 1.53-3.36), 40 to 49 years (OR, 1.65; 95% CI, 1.11-2.44), and 50 to 59 years (OR, 1.47; 95% CI, 1.01-2.12); educational level at primary school (OR, 1.99; 95% CI, 1.51-2.64) and upper secondary school (OR, 1.57; 95% CI, 1.25-1.96); former smoking (OR, 2.37; 95% CI, 1.73-3.24); and current smoking (OR, 18.10; 95% CI, 14.19-23.09). All respiratory symptoms were most common among dual users and former smokers and nonsmokers who used e-cigarettes.

**Conclusions and Relevance:**

Use of e-cigarettes was most common among smokers, and dual users had the highest prevalence of respiratory symptoms. On a population level, this study indicates that the present use of e-cigarettes does not adequately serve as a smoking cessation tool.

## Key Points

**Question:**

Who uses electronic cigarettes (e-cigarettes), and is there an association between e-cigarette use and respiratory symptoms?

**Findings:**

In a random sample of more than 30 000 Swedish adults, e-cigarette use was most common among current smokers, and the prevalence of respiratory symptoms was highest among the current smokers who also used e-cigarettes.

**Meaning:**

Longitudinal studies will be essential to further determine the long-term health effects of e-cigarette use and whether in dual users it increases the burden of respiratory conditions or encourages sustainable smoking cessation.

## Teaser

This cross-sectional study uses survey data from the Obstructive Lung Disease in Northern Sweden (OLIN) and West Sweden Asthma Study (WSAS) surveys to determine the prevalence of electronic cigarette (e-cigarette) use and investigate the association of e-cigarette use with smoking habits, demographic characteristics, and respiratory symptoms in Swedish adults.

## Introduction

During the last 30 years, the proportion of smokers has steadily decreased in Sweden, which has contributed to a decreased prevalence of respiratory symptoms and chronic obstructive pulmonary disease (COPD) among adults,^1,2^ less exposure to environmental tobacco smoke,^3^ and a decrease in lung cancer mortality among men.^4^ This positive public health trend may now be threatened as the tobacco industry continues to develop and market new nicotine delivery devices, including electronic cigarettes (e-cigarettes). Since their introduction to the market 10 years ago, e-cigarettes have rapidly become a billion-dollar industry; however, globally the content, sales, and marketing remain largely unregulated and the possible adverse health effects have yet to be established or refuted.^5,6,7^

There is an ongoing and often heated debate about whether e-cigarettes are the solution to the tobacco epidemic or a potential danger.^6,7^ Those in favor argue that e-cigarettes help smokers quit smoking conventional cigarettes, that they play an important role in tobacco harm reduction, and that they contribute to a reduction of exposure to environmental tobacco smoke.^5,8,9^ Others argue that switching to e-cigarettes may allow smokers to maintain their smoking behavior while reducing their exposure to several of the hazardous substances of a conventional cigarette.^10^ Those against argue that e-cigarettes prolong or have no effect on smoking cessation, that they do have an adverse effect on respiratory health, and that they encourage dual use: smoking both electronic and combustible cigarettes and choosing a favored product based on environment and occasion, thus effectively supplementing their habit.^11,12^ Furthermore, it has been shown that e-cigarettes may serve as a gateway to smoking conventional cigarettes among nonsmoking adolescents.^13^

Exposure studies performed in humans and animals indicate that e-cigarettes may have respiratory and acute vascular effects.^14,15^ However, observational studies on the health effects of e-cigarettes are lacking. According to a systematic review, the results were inconsistent, and there were conflicts of interest with the tobacco or e-cigarette industry in 34% of the studies.^16^ It will take time to fully establish the long-term effects of exposure to e-cigarettes as they are a completely novel product. However, as of today there are some cross-sectional studies that have found associations between e-cigarette use and respiratory symptoms as well as increased prevalence and severity of symptoms in asthma among adolescents.^17,18^

The e-cigarette industry portrays e-cigarettes to the regulatory, health, and scientific communities as a substitute for conventional cigarettes and a means of smoking cessation. However, their efficacy as a smoking cessation tool has yet to be established, and, paradoxically, much of the marketing is devised to appeal to adolescents and nonsmokers.^19^ The prevalence of regular use of e-cigarettes is estimated to still be relatively low in the general population, with a rate of 1% to 4%.^20,21,22,23^ Therefore, large representative population-based studies are needed to examine factors associated with e-cigarette use in the general population. Our hypothesis was that e-cigarettes are used as marketed by the e-cigarette industry and thereby most common among former smokers. The aim was to estimate the prevalence of e-cigarette use in relation to smoking habits and other demographic factors among Swedish adults as well as to study the association with respiratory symptoms.

## Methods

### Study Sample and Procedure

In 2016 the Obstructive Lung Disease in Northern Sweden (OLIN) study and the West Sweden Asthma Study (WSAS) conducted postal questionnaire surveys in random samples of the adult population aged 20 to 75 years in 2 large geographical areas of Sweden, the counties of Norrbotten in the north and Västra Götaland in the southwest.^24,25^ These cross-sectional studies were performed using identical methods and during the same time of year with a starting point in January. After 3 reminders had been sent, there were 6519 participants in OLIN and 23 753 participants in WSAS. Using the American Association for Public Opinion Research (AAPOR) reporting guideline, we calculated a response rate of 56.4% for OLIN and 50.1% for WSAS. The OLIN and WSAS studies were approved by the regional ethical review boards in Umeå, Sweden, and Gothenburg, Sweden, respectively. All participants gave their written informed consent to participate in the study as they returned the postal questionnaire.

### Questionnaire and Definitions

The same validated questionnaire was used by OLIN and WSAS. It included questions about respiratory symptoms during the last 12 months, smoking habits, and current e-cigarette use, as well as questions about demographic characteristics such as age, sex, and educational level categorized into primary school, upper secondary school, or higher education. The questionnaire has previously been described in detail.^1,24,26^ Current smokers were defined as those who gave an affirmative answer to the question “Do you smoke?” Former smokers were defined as those who gave an affirmative answer to the question “Have you been a smoker but have stopped smoking more than 1 year ago?” Nonsmokers were those who gave negative answers to the questions “Do you smoke?” and “Have you been a smoker but have stopped smoking more than 1 year ago?” Electronic cigarette use was defined as answering “sometimes” or “daily” to the question “Do you use e-cigarettes?” Those answering “never” were classified as having no e-cigarette use. Dual use was defined as being both a current smoker and an e-cigarette user. Respiratory symptoms were defined by affirmative answers to the following questions: for long-standing cough, “Have you had long-standing cough during the last year?”; for sputum production, “Do you usually have phlegm when coughing, or do you have phlegm in your chest that is difficult to bring up?”; for chronic productive cough, “Do you bring up phlegm on most days during periods of at least 3 months?” and “Have you had such periods during at least 2 successive years?”; for any wheeze, “Have you at any time during the last 12 months had wheezing or whistling in your chest?”; for recurrent wheeze, “Do you usually have wheezing, whistling, or a noisy sound in your chest when breathing?”; and for any respiratory symptoms, an affirmative answer to any of the questions on respiratory symptoms.

### Statistical Analysis

Analyses were performed using the SPSS Statistics software version 24 (IBM). Differences in proportions between groups were analyzed by the χ^2^ test, or Mantel-Haenszel test for trend when there were more than 2 categories. A 2-sided value of *P* < .05 was considered statistically significant. For questions on respiratory symptoms, missing answers to individual questions (<2%) were regarded as negative responses. Missing answers to the questions about educational level (1%), smoking habits (1%), and e-cigarette use (13%) were regarded as missing and excluded from the analyses. Factors significantly associated with e-cigarette use in bivariate analyses were included in multivariable logistic regression models with the results expressed as odds ratios (ORs) with 95% confidence intervals. In the analyses of the association between e-cigarette use and respiratory symptoms, each of the groups (nonsmokers, former smokers, and current smokers) were further categorized into e-cigarette users and non–e-cigarette users. Nonsmokers without e-cigarette use were used as the reference in the regression analysis.

## Results

### Demographic Characteristics

Basic characteristics of the participants in WSAS and OLIN are presented in Table 1. Of 30 272 participants (16 325 women [53.9%]), 3897 (12.9%) were aged 20 to 29 years; 4242 (14.0%), 30 to 39 years; 5082 (16.8%), 40 to 49 years; 6052 (20.0%), 50 to 59 years; 6628 (21.9%), 60 to 69 years; and 4371 (14.4%), 70 to 75 years. Among the participants in WSAS there were more women, a higher proportion of individuals in the younger age groups, and more respondents with a higher educational level. Electronic cigarette use was more common in WSAS than in OLIN, while the smoking habits were similar in the 2 surveys. Overall, 529 participants (2.0%) used e-cigarettes, 3694 (12.3%) were current smokers, and 7305 (24.4%) were former smokers. Electronic cigarette use was more common among men (275 of 12 347 [2.2%; 95% CI, 2.0%-2.5%]) than women (254 of 14 022 [1.8%; 95% CI, 1.6%-2.0%]), while current smoking was more common among women (2063 of 16 167 [12.8%; 95% CI, 12.3%-13.3%]) than men (1631 of 13 792 [11.8%; 95% CI, 11.3%-12.3%]).

**Table 1.** Characteristics of Participants in 2 Questionnaire Surveys by Survey and Sex

Characteristic	All (N = 30 272)	Survey			Sex		
		OLIN (n = 6519)	WSAS (n = 23 753)	*P* Value	Men (n = 13 947)	Women (n = 16 325)	*P* Value
Sex, No. (%)							
Men	13 947 (46.1)	3102 (47.6)	10 845 (45.7)	.006			
Women	16 325 (53.9)	3417 (52.4)	12 908 (54.3)				
Age, y, No. (%)				<.001			
20-29	3897 (12.9)	830 (12.7)	3067 (12.9)		1657 (11.9)	2240 (13.7)	<.001
30-39	4242 (14.0)	724 (11.1)	3518 (14.8)		1878 (13.5)	2364 (14.5)	
40-49	5082 (16.8)	1001 (15.4)	4081 (17.2)		2290 (16.4)	2792 (17.1)	
50-59	6052 (20.0)	1311 (20.1)	4741 (20.0)		2749 (19.7)	3303 (20.2)	
60-69	6628 (21.9)	1600 (24.5)	5028 (21.2)		3197 (22.9)	3431 (21.0)	
70-75	4371 (14.4)	1053 (16.2)	3318 (14.0)		2176 (15.6)	2195 (13.4)	
Educational level, No. (%)							
Primary school	5027 (16.8)	1083 (17.0)	3944 (16.8)	<.001	2476 (18.0)	2251 (15.9)	<.001
Upper secondary school	11 911 (39.9)	3074 (48.3)	8837 (37.6)		6177 (44.8)	5734 (35.7)	
Higher education	12 914 (43.3)	2209 (34.7)	10 705 (45.6)		5132 (37.2)	7782 (48.4)	
Smoking, No. (%)							
None	18 960 (63.3)	4096 (64.7)	14 864 (62.9)	.06	8746 (63.4)	10 214 (63.2)	.15
Former	7305 (24.4)	1464 (23.1)	5841 (24.7)		3415 (24.8)	3890 (24.1)	
Current	3694 (12.3)	775 (12.2)	2919 (12.4)		1631 (11.8)	2063 (12.8)	
E-cigarette use, No. (%)	529 (2.0)	73 (1.3)	456 (2.2)	<.001	275 (2.2)	254 (1.8)	.02

Abbreviations: E-cigarette, electronic cigarette; OLIN, Obstructive Lung Disease in Northern Sweden; WSAS: West Sweden Asthma Study.

### Electronic Cigarette Use in Relation to Smoking Habits

Among smokers, 350 of 3566 (9.8%; 95% CI, 8.8%-10.8%) used e-cigarettes compared with 79 of 6875 former smokers (1.1%; 95% CI, 0.9%-1.3%) and 96 of 15 832 nonsmokers (0.6%; 95% CI, 0.5%-0.7%) (*P* < .001). This pattern was more pronounced in the younger age groups: among smokers aged 20 to 29 years, 79 of 583 (13.6%; 95% CI, 10.8%-16.4%) used e-cigarettes. Among smokers aged 30 to 39 years, 53 of 415 (12.8%; 95% CI, 9.6%-16.0%) used e-cigarettes. Among e-cigarette users who answered the survey question about cigarette-smoking habits (n = 525), 350 (66.7%; 95% CI, 62.7%-70.7%) were current smokers, 79 (15.0%; 95% CI, 11.9%-18.1%) were former smokers, and 96 (18.3%; 95% CI, 15.0%-21.6%) were nonsmokers (*P* < .001 for trend). The proportion of nonsmokers among e-cigarette users was significantly higher among men (61 of 7478 [22.3%; 95% CI, 21.4%-23.2%]) than women (35 of 8354 [13.9%; 95% CI, 13.2%-14.6%]) (*P* = .04).

### Factors Associated With Electronic Cigarette Use

**Figure 1.**
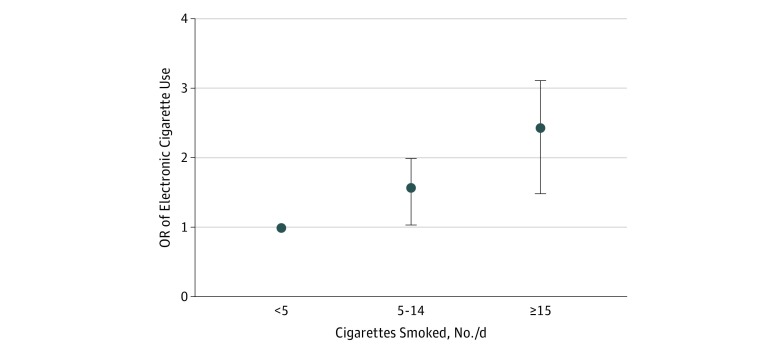
Electronic Cigarette Use in Relation to Number of Conventional Cigarettes per Day Among Smokers The odds ratio (OR) of electronic cigarette use increased as the number of cigarettes smoked per day increased. The ORs are adjusted for sex, age group, educational level, and survey. Error bars indicate 95% CI.

In the regression analysis, e-cigarette use was significantly related to male sex (OR, 1.35; 95% CI, 1.12-1.62); the age groups 20 to 29 years (OR, 2.77; 95% CI, 1.90-4.05), 30 to 39 years (OR, 2.27; 95% CI, 1.53-3.36), 40 to 49 years (OR, 1.65; 95% CI, 1.11-2.44), and 50 to 59 years (OR, 1.47; 95% CI, 1.01-2.12); educational level at primary school (OR, 1.99; 95% CI, 1.51-2.64) and upper secondary school (OR, 1.57; 95% CI, 1.25-1.96); former smoking (OR, 2.37; 95% CI, 1.73-3.24); and current smoking (OR, 18.10; 95% CI, 14.19-23.09) (Table 2). Furthermore, the corresponding analysis was performed among smokers only, but also including the number of cigarettes smoked per day. The OR for e-cigarette use increased with increasing number of cigarettes smoked per day (Figure 1). The prevalence of e-cigarette use was 13.8% (99 of 718 participants [95% CI, 11.3%-16.3%]) among those smoking 15 or more cigarettes per day, 9.7% (144 of 1489 participants [95% CI, 8.2%-11.2%]) among those smoking 5 to 14 cigarettes per day, and 7.5% (96 of 1273 participants [95% CI, 6.1%-8.9%]) among those smoking fewer than 5 cigarettes per day (*P* < .001 for trend).

**Table 2.** Factors Associated With Electronic Cigarette Use, Analyzed by Multivariable Logistic Regression

Factor	OR (95% CI)
Sex	
Men	1.35 (1.12-1.62)
Women	1 [Reference]
Age, y	
20-29	2.77 (1.90-4.05)
30-39	2.27 (1.53-3.36)
40-49	1.65 (1.11-2.44)
50-59	1.47 (1.01-2.12)
60-69	0.96 (0.66-1.39)
70-75	1 [Reference]
Educational level	
Primary school	1.99 (1.51-2.64)
Upper secondary school	1.57 (1.25-1.96)
Higher education	1 [Reference]
Smoking habits	
None	1 [Reference]
Former	2.37 (1.73-3.24)
Current	18.10 (14.19-23.09)
Study	
OLIN	1 [Reference]
WSAS	1.65 (1.27-2.15)

Abbreviations: OLIN, Obstructive Lung Disease in Northern Sweden; OR, odds ratio; WSAS, West Sweden Asthma Study.

### Respiratory Symptoms in Relation to Electronic Cigarette Use and Smoking

**Figure 2.**
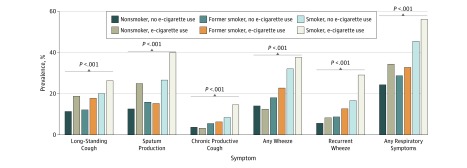
Prevalence of Respiratory Symptoms by Smoking Habits and Electronic Cigarette (E-cigarette) Use All respiratory symptoms were most common among dual users. Respiratory symptoms were more common among e-cigarette users among both former smokers and nonsmokers. The Mantel-Haenszel test for trend was used to generate *P* values.

All respiratory symptoms were most common among dual users. Furthermore, respiratory symptoms were generally more common among e-cigarette users among both former smokers and nonsmokers (Figure 2). In a regression analysis adjusted for sex, age group, survey, and educational level, having any respiratory symptom was significantly associated with dual use (OR, 4.03; 95% CI, 3.23-5.02), smoking only (OR, 2.55; 95% CI, 2.36-2.77), and former smoking without e-cigarette use (OR, 1.27; 95% CI, 1.19-1.36), while former smoking with e-cigarette use (OR, 1.47; 95% CI, 0.91-2.37) and nonsmoking with e-cigarette use (OR, 1.46; 95% CI, 0.93-2.29) did not reach statistical significance (Table 3). Corresponding analyses for each of the respiratory symptoms are presented in eTables 1 through 5 in the Supplement. In a stratified analysis among smokers adjusting for number of cigarettes smoked per day, having any respiratory symptoms remained significantly associated with e-cigarette use (OR, 1.45; 95% CI, 1.15-1.84).

**Table 3.** Participants Reporting Any Respiratory Symptoms in Relation to Smoking Habits and E-cigarette Use, Analyzed by Logistic Regression

Smoking and E-cigarette Use	OR (95% CI)	
	Unadjusted	Adjusted^a^
Nonsmoker		
No e-cigarette use	1 [Reference]	1 [Reference]
E-cigarette use	1.62 (1.06-2.47)	1.46 (0.93-2.29)
Former smoker		
No e-cigarette use	1.25 (1.17-1.33)	1.27 (1.19-1.36)
E-cigarette use	1.52 (0.95-2.43)	1.47 (0.91-2.37)
Smoker		
No e-cigarette use	2.59 (2.40-2.81)	2.55 (2.36-2.77)
E-cigarette use	3.98 (3.21-4.93)	4.03 (3.23-5.02)

Abbreviations: E-cigarette, electronic cigarette; OR, odds ratio.

^a^ Adjusted for sex, age group, educational level, and survey.

### Comparison of the Invited Sample vs Responders

Of the invited sample in OLIN, 51.9% (95% CI, 51.1%-52.9%) were men vs 47.5% (95% CI, 46.8%-49.2%) among the responders (*P* < .001). The age distribution of the invited participants vs responders was as follows: 20 to 29 years, 20% vs 13%; 30 to 39 years, 15% vs 11%; 40 to 49 years, 17% vs 15%; 50 to 59 years, 18% vs 20%; 60 to 69 years, 19% vs 25%; and 70 to 75 years, 12% vs 16%. Of the invited sample in WSAS, 50.3% (95% CI, 49.5%-50.5%) were men vs 45.7% (95% CI, 45.4%-46.6%) among the responders (*P* < .001). The age distribution of those invited vs those who responded was as follows: 20 to 29 years, 21% vs 13%; 30 to 39 years, 19% vs 15%; 40 to 49 years, 19% vs 17%; 50 to 59 years, 18% vs 20%; 60 to 69 years, 16% vs 21%; and 70 to 75 years, 7% vs 14%.

## Discussion

In this cross-sectional study of random samples of the Swedish population, we found that the prevalence of e-cigarette use was 2%. Factors related to e-cigarette use were male sex, younger age, lower educational level, and smoking. Electronic cigarette use was most common among current smokers, and these dual users had the highest prevalence of respiratory symptoms.

The 2% prevalence of current e-cigarette use in our study is slightly lower than, but still in line with, other large random samples of the population in Europe^20^ and North America.^21,22,23^ Until recently there have been strict regulations regarding store sales in Sweden; therefore, e-cigarettes have mainly been purchased online. Online sales as well as targeted advertising tend to skew use toward a younger demographic.^22^ Not surprisingly, and in correspondence with other studies,^20,23,27^ we found that e-cigarette use was more common in younger groups.

It has been proposed that e-cigarettes may serve as a gateway to smoking.^13,28^ Therefore, our finding that 1 of 5 e-cigarette users were nonsmokers is noteworthy and raises concern for their future risk of becoming smokers. Furthermore, this group is also at risk of becoming addicted to nicotine, a potent toxicant that has been suggested to accelerate atherosclerosis, thus increasing the risk of cardiovascular disease.^29^ On the other hand, this also highlights the need to study health effects of e-cigarettes as a separate entity instead of simply correlating all findings to known effects of conventional cigarettes. Nevertheless, the highest proportion of e-cigarette users were found among current smokers, in accordance with many other studies.^21,22,23,27^ In Sweden, smoking is more common among women and individuals older than 50 years,^25^ whereas e-cigarette use seems to be more common among men and individuals younger than 40 years. Thus, e-cigarettes seem to appeal to new target groups and may increase the likelihood of future smoking among formerly low-risk groups.

Our hypothesis that e-cigarette use would be most common among former smokers could not be verified. Instead, e-cigarette use was most common among smokers, particularly among those smoking a higher number of conventional cigarettes per day. Possible explanations for dual use are that this group includes smokers who want to quit and may have just initiated e-cigarette use, thus becoming dual users for a period while trying to attain smoking cessation. Another explanation may be that smokers initiate e-cigarette use to augment their smoking habits in the increasing number of situations or environments where conventional smoking has been banned, for instance, at restaurants, on public transportation, or in other public spaces. On the other hand, it may simply be that e-cigarettes are not helping them to quit. Their efficacy as a smoking cessation tool is under heavy debate: a recent meta-analysis showed lower odds of smoking cessation among e-cigarette users than nonusers,^19^ whereas a systematic review found most studies demonstrated a positive association between e-cigarette use and smoking cessation, although the quality of evidence was assessed as low.^11^ On a population level, our study seems to indicate that the present use of e-cigarettes does not adequately serve as a smoking cessation tool.

Respiratory symptoms were in general more common among e-cigarette users, mainly among current smokers but also among nonsmokers and former smokers. When adjusted for sex, age group, survey, and educational level, the association with respiratory symptoms remained significant for dual use, smoking only, and former smoking without e-cigarette use but not for former smoking with e-cigarette use. This contrasts with 2 other studies that found a higher risk of bronchitis symptoms among e-cigarette users and former smokers but not in current smokers.^12,18^ However, these studies were performed solely among adolescents and, to our knowledge, there are comparatively few population-based studies on the association between e-cigarette use and respiratory symptoms among adults. Previously published studies are mainly experimental and laboratory studies in small selected samples. Nevertheless, growing evidence points toward e-cigarettes having adverse pulmonary and vascular effects.^14,15,30^ Because e-cigarettes are a relatively novel product, it will take time for the long-term health effects of e-cigarette use in humans to be identified. Until then regulators and the medical community should err on the side of caution. Longitudinal studies are needed to determine what role e-cigarettes will play in the tobacco epidemic: whether e-cigarette use will increase the burden of respiratory conditions or contribute to durable smoking cessation results.

The most important tactics of tobacco control include preventing adolescents from initiating tobacco use, helping smokers quit, and minimizing involuntary passive exposure to environmental tobacco smoke. Harm reduction for conventional cigarettes could be achieved by a switch to alternative tobacco or nicotine products. A similar debate and controversy as that surrounding the introduction of e-cigarettes occurred in the 1950s and 1960s as well, when filtered and low-tar cigarettes were introduced.^31^ At the time, health authorities advised physicians to encourage their smoking patients to switch to these new products, while others argued that this discouraged smokers from quitting and did not reduce the health risks associated with smoking, which was later demonstrated to be the case. Thus, similar debates have been ongoing for more than 50 years, with only the type of nicotine-containing product being different. Many smokers express a desire to quit and therefore seek products that will reduce the risk to their health^32^ and at the same time satisfy their habitual needs and nicotine addiction. It may be that e-cigarettes fulfill these demands, as the first generation of devices resembled a conventional cigarette, they have variable nicotine content, and they have been perceived as a safer alternative.^8^ Compared with conventional cigarette smoke, the levels of certain toxic compounds found in e-cigarette vapor have been shown to be considerably lower.^10^ However, it is currently impossible for e-cigarette users to know exactly what they are inhaling because of the lack of regulation, which allows for the content of e-cigarette liquids to vary greatly and has been shown to be inconsistent with the labeling.^33,34^ For instance, 7 of 10 products labeled nicotine free by the manufacturer did in fact contain nicotine.^34^ Furthermore, the vapor has been demonstrated to contain high levels of heavy metals and toxic chemicals, which are known airway irritants.^34,35^ Thus, even though e-cigarettes are noncombustible and do not contain tar, they do still contain chemicals and carcinogenic compounds that may have adverse health effects. Therefore, the medical community needs to be careful when recommending e-cigarettes to patients as a smoking cessation method or as a safer alternative to conventional cigarettes, especially as their efficacy as a smoking cessation method is still ambiguous.^19^

### Limitations

The strengths of this study include the large, randomly selected sample of the population and a well-validated questionnaire. The identical questionnaire used in OLIN and WSAS enabled pooling of data to form a study sample with enough statistical power to perform analyses of e-cigarette use. However, despite the large sample size, adjusted analyses among e-cigarette users between former smokers and nonsmokers were not possible because of a relatively low prevalence of e-cigarette use in the total sample population. Because of the nature of a cross-sectional study, we are limited in the ability to draw conclusions about causality. The survey response rates were 50.1% and 56.4%, which may have caused selection bias and lack of representativeness. Several studies of nonresponders in epidemiological studies of respiratory diseases have shown that men, younger individuals, and smokers are less likely to respond.^36,37,38,39^ Even though WSAS has previously demonstrated that nonresponse in a postal questionnaire survey did not affect the risk estimates,^39^ the lower participation rate among younger men and smokers may have resulted in less robust prevalence estimates and an underestimation of e-cigarette users in the population.

## Conclusions

Electronic cigarette use was most common among smokers, and dual users had the highest prevalence of respiratory symptoms. Electronic cigarette use was associated with male sex, younger age, lower educational level, and both former and current smoking. On a population level, our study seems to indicate that the present use of e-cigarettes does not adequately serve as a smoking cessation tool. Longitudinal studies will be essential to further determine the long-term health effects of e-cigarette use and whether in dual users it will increase the burden of respiratory conditions or encourage sustainable smoking cessation.

## Notes

Supplement. **eTable 1.** Long-standing Cough in Relation to Smoking Habits and E-Cigarette Use, Analyzed by Logistic Regression, Presented as Unadjusted and Adjusted for Sex, Age Group, Educational Level, and Study Area and Expressed as Odds Ratios (OR) With 95% Confidence Intervals (CI). **eTable 2.** Sputum Production in Relation to Smoking Habits and E-Cigarette Use, Analyzed by Logistic Regression, Presented as Unadjusted and Adjusted for Sex, Age Group, Educational Level, and Study Area and Expressed as Odds Ratios (OR) With 95% Confidence Intervals (CI). **eTable 3.** Chronic Productive Cough in Relation to Smoking Habits and E-Cigarette Use, Analyzed by Logistic Regression, Presented as Unadjusted and Adjusted for Sex, Age Group, Educational Level, and Study Area and Expressed as Odds Ratios (OR) With 95% Confidence Intervals (CI). **eTable 4.** Any Wheeze in Relation to Smoking Habits and E-Cigarette Use, Analyzed by Logistic Regression, Presented as Unadjusted and Adjusted For Sex, Age Group, Educational Level, and Study Area and Expressed as Odds Ratios (OR) With 95% Confidence Intervals (CI). **eTable 5.** Recurrent Wheeze in Relation to Smoking Habits and E-Cigarette Use, Analyzed by Logistic Regression, Presented as Unadjusted and Adjusted for Sex, Age Group, Educational Level, and Study Area and Expressed as Odds Ratios (OR) With 95% Confidence Intervals (CI).
