# Supplementary material for: Association of Electronic Cigarette Use With Smoking Habits, Demographic Factors, and Respiratory Symptoms
Source: JAMA Netw Open. 2018 Jul 20;1(3):e180789. doi: 10.1001/jamanetworkopen.2018.0789 (PMC6324524; doi:10.1001/jamanetworkopen.2018.0789)
Supplement: Supplement. — eTable 1. Long-standing Cough in Relation to Smoking Habits and E-Cigarette Use, Analyzed by Logistic Regression, Presented as Unadjusted and Adjusted for Sex, Age Group, Educational Level, and Study Area and Expressed as Odds Ratios (OR) With 95% Confidence Intervals (CI). eTable 2. Sputum Production in Relation to Smoking Habits and E-Cigarette Use, Analyzed by Logistic Regression, Presented as Unadjusted and Adjusted for Sex, Age Group, Educational Level, and Study Area and Expressed as Odds Ratios (OR) With 95% Confidence Intervals (CI). eTable 3. Chronic Productive Cough in Relation to Smoking Habits and E-Cigarette Use, Analyzed by Logistic Regression, Presented as Unadjusted and Adjusted for Sex, Age Group, Educational Level, and Study Area and Expressed as Odds Ratios (OR) With 95% Confidence Intervals (CI). eTable 4. Any Wheeze in Relation to Smoking Habits and E-Cigarette Use, Analyzed by Logistic Regression, Presented as Unadjusted and Adjusted For Sex, Age Group, Educational Level, and Study Area and Expressed as Odds Ratios (OR) With 95% Confidence Intervals (CI). eTable 5. Recurrent Wheeze in Relation to Smoking Habits and E-Cigarette Use, Analyzed by Logistic Regression, Presented as Unadjusted and Adjusted for Sex, Age Group, Educational Level, and Study Area and Expressed as Odds Ratios (OR) With 95% Confidence Intervals (CI). [file jamanetwopen-1-e180789-s001.pdf]

Supplementary Online Content

Hedman L, Backman H, Stridsman C, et al. Association of electronic cigarette use with smoking habits, demographic factors, and respiratory symptoms. *JAMA Netw Open*. 2018;1(3):e180789. doi:10.1001/jamanetworkopen.2018.0789

eTable 1. Long-standing Cough in Relation to Smoking Habits and E-Cigarette Use, Analyzed by Logistic Regression, Presented as Unadjusted and Adjusted for Sex, Age Group, Educational Level, and Study Area and Expressed as Odds Ratios (OR) With 95% Confidence Intervals (CI).

eTable 2. Sputum Production in Relation to Smoking Habits and E-Cigarette Use, Analyzed by Logistic Regression, Presented as Unadjusted and Adjusted for Sex, Age Group, Educational Level, and Study Area and Expressed as Odds Ratios (OR) With 95% Confidence Intervals (CI).

eTable 3. Chronic Productive Cough in Relation to Smoking Habits and E-Cigarette Use, Analyzed by Logistic Regression, Presented as Unadjusted and Adjusted for Sex, Age Group, Educational Level, and Study Area and Expressed as Odds Ratios (OR) With 95% Confidence Intervals (CI).

eTable 4. Any Wheeze in Relation to Smoking Habits and E-Cigarette Use, Analyzed by Logistic Regression, Presented as Unadjusted and Adjusted for Sex, Age Group, Educational Level, and Study Area and Expressed as Odds Ratios (OR) With 95% Confidence Intervals (CI).

eTable 5. Recurrent Wheeze in Relation to Smoking Habits and E-Cigarette Use, Analyzed by Logistic Regression, Presented as Unadjusted and Adjusted for Sex, Age Group, Educational Level, and Study Area and Expressed as Odds Ratios (OR) With 95% Confidence Intervals (CI).

This supplementary material has been provided by the authors to give readers additional information about their work.

Number of eTables: 5

eTable 1-5: Unadjusted and adjusted logistic regression analyses. Dependent variables: longstanding cough, sputum production, chronic productive cough, recurrent wheeze and any wheeze. Independent variables: smoking habits and e-cigarette use, sex, age-group, educational level and study area.

© 2018 Hedman L et al. *JAMA Network Open.*

eTable 1. Long-standing cough in relation to smoking habits and e-cigarette use, analyzed by logistic regression, presented as unadjusted and adjusted for sex, age group, educational level, and study area and expressed as odds ratios (OR) with 95% confidence intervals (CI).

	Unadjusted			Adjusted	
	**OR**	**(95% CI)**		**OR**	**(95% CI)**
Non-smoker, No e-cigarette use	1.00			1.00	
Non-smoker, E-cigarette use	1.81	(1.08-3.02)		1.60	(0.92-2.80)
Former smoker, No e-cigarette use	1.09	(1.00-1.19)		1.10	(1.00-1.20)
Former smoker, E-cigarette use	1.69	(0.95-3.01)		1.57	(0.86-2.86)
Smoker, No e-cigarette use	1.96	(1.78-2.17)		1.93	(1.74-2.13)
Smoker, E-cigarette use	2.79	(2.19-3.56)		2.83	(2.21-3.63)

© 2018 Hedman L et al. *JAMA Network Open.*

eTable 2. Sputum production in relation to smoking habits and e-cigarette use, analyzed by logistic regression, presented as unadjusted and adjusted for sex, age group, educational level, and study area and expressed as odds ratios (OR) with 95% confidence intervals (CI).

	Unadjusted			Adjusted	
	**OR**	**(95% CI)**		**OR**	**(95% CI)**
Non-smoker, No e-cigarette use	1.00			1.00	
Non-smoker, E-cigarette use	2.33	(1.46-3.71)		1.96	(1.18-3.24)
Former smoker, No e-cigarette use	1.32	(1.22-1.43)		1.28	(1.18-1.39)
Former smoker, E-cigarette use	1.25	(0.68-2.32)		1.12	(0.59-2.12)
Smoker, No e-cigarette use	2.54	(2.32-2.79)		2.44	(2.22-2.68)
Smoker, E-cigarette use	4.66	(3.74-5.80)		4.52	(3.61-5.65)

© 2018 Hedman L et al. *JAMA Network Open.*

eTable 3. Chronic productive cough in relation to smoking habits and e-cigarette use, analyzed by logistic regression, presented as unadjusted and adjusted for sex, age group, educational level, and study area and expressed as odds ratios (OR) with 95% confidence intervals (CI).

	**Unadjusted**			**Adjusted**	
	**OR**	**(95% CI)**		**OR**	**(95% CI)**
Non-smoker, No e-cigarette use	1.00			1.00	
Non-smoker, E-cigarette use	0.87	(0.28-2.77)		0.62	(0.15-2.51)
Former smoker, No e-cigarette use	1.57	(1.37-1.79)		1.36	(1.19-1.57)
Former smoker, E-cigarette use	1.83	(0.74-4.55)		1.73	(0.69-4.30)
Smoker, No e-cigarette use	2.47	(2.13-2.88)		2.28	(1.96-2.67)
Smoker, E-cigarette use	4.62	(3.40-6.30)		4.43	(3.22-6.08)

© 2018 Hedman L et al. *JAMA Network Open.*

eTable 4. Any wheeze in relation to smoking habits and e-cigarette use, analyzed by logistic regression, presented as unadjusted and adjusted for sex, age group, educational level, and study area and expressed as odds ratios (OR) with 95% confidence intervals (CI).

	**Unadjusted**			**Adjusted**	
	**OR**	**(95% CI)**		**OR**	**(95% CI)**
Non-smoker, No e-cigarette use	1.00			1.00	
Non-smoker, E-cigarette use	0.88	(0.48-1.61)		0.87	(0.46-1.64)
Former smoker, No e-cigarette use	1.36	(1.26-1.47)		1.37	(1.27-1.49)
Former smoker, E-cigarette use	1.82	(1.07-3.08)		1.73	(1.01-2.97)
Smoker, No e-cigarette use	2.90	(2.66-3.16)		2.83	(2.59-3.10)
Smoker, E-cigarette use	3.72	(2.99-4.64)		3.86	(3.08-4.83)

© 2018 Hedman L et al. *JAMA Network Open.*

eTable 5. Recurrent wheeze in relation to smoking habits and e-cigarette use, analyzed by logistic regression, presented as unadjusted and adjusted for sex, age group, educational level, and study area and expressed as odds ratios (OR) with 95% confidence intervals (CI).

	Unadjusted			Adjusted	
	OR	(95% CI)		OR	(95% CI)
Non-smoker, No e-cigarette use	1.00			1.00	
Non-smoker, E-cigarette use	1.53	(0.74-3.17)		1.46	(0.67-3.18)
Former smoker, No e-cigarette use	1.62	(1.45-1.80)		1.48	(1.32-1.65)
Former smoker, E-cigarette use	2.44	(1.26-4.76)		2.16	(1.07-4.37)
Smoker, No e-cigarette use	3.36	(2.99-3.77)		3.02	(2.68-3.41)
Smoker, E-cigarette use	6.94	(5.45-8.82)		6.88	(5.37-8.80)

© 2018 Hedman L et al. *JAMA Network Open.*
